# Cutaneous Squamous Cell Carcinoma Masquerading as a Verruca: Case Report and Literature Review of Coexisting Wart and Invasive Squamous Cell Carcinoma on the Hand

**DOI:** 10.7759/cureus.32408

**Published:** 2022-12-11

**Authors:** Philip R Cohen, Christof P Erickson, Antoanella Calame

**Affiliations:** 1 Dermatology, University of California, Davis Medical Center, Sacramento, USA; 2 Dermatology, Compass Dermatopathology, San Diego, USA; 3 Dermatology/Dermatopathology, Compass Dermatopathology, San Diego, USA; 4 Dermatology, Scripps Memorial Hospital, La Jolla, USA

**Keywords:** wart, vulgaris, verruca, squamous, papillomavirus, human, hand, cutaneous, cell, carcinoma

## Abstract

A verruca is a human papillomavirus-associated infection of the mucosal or cutaneous epithelium. Cutaneous squamous cell carcinoma is an invasive skin cancer that commonly occurs on sun-exposed locations. Human papillomavirus infection has also been demonstrated to be a cocarcinogen, along with ultraviolet radiation, in the pathogenesis of cutaneous squamous cell carcinoma. A 63-year-old man presented with a verrucous nodule of nine months duration on his dorsal left hand. The clinical differential diagnosis included a wart and a small punch biopsy of the lesion showed a verruca. The lesion continued to enlarge and the possibility of a squamous cell carcinoma was considered. A second larger shave biopsy of the residual lesion was performed and the microscopic evaluation revealed not only a benign verruca at the lateral portion of the nodule but also an invasive squamous cell carcinoma in the center of the lesion. We hypothesize that the patient’s human papillomavirus-associated wart may have contributed to the development of his cutaneous squamous cell carcinoma. Therefore, in an individual with a clinically suspected or biopsy-confirmed wart that persists despite lesion-directed treatment, additional evaluation of the lesion should be considered to assess whether an alternative or concurrent tumor, such as a cutaneous squamous cell carcinoma, is present.

## Introduction

A verruca, also referred to as a wart, is caused by human papillomavirus. The morphology of these lesions is influenced by the viral serotype, location, and immune status of the patient. Therefore, there are several clinical variants of this contagious viral infection and each is associated with one or more human papillomavirus strains. The types of verrucae include butcher’s warts, common warts (such as filiform warts and verruca vulgaris), cystic warts, flat (plane) warts, genital warts, oral warts, and palmar and plantar warts [1,2].

Cutaneous squamous cell carcinoma is a frequently occurring form of skin cancer second only to basal cell carcinoma. Sun-exposed areas, such as the head, neck, and extensor surfaces of the hands and arms, are the most common tumor locations. Tumorigenesis of cutaneous squamous cell carcinoma is associated with risk factors such as cumulative sun exposure and immunosuppression; in addition, human papillomavirus infection has been linked to the carcinogenesis of this skin cancer [3-6].

Squamous cell carcinoma can masquerade clinically as a verruca; in addition, the tumor can demonstrate the presence of human papillomavirus or pathologic features of a wart or both [5,6]. A 63-year-old man developed a verrucous nodule on his hand. The clinical differential diagnosis included a wart and a small biopsy showed a verruca; however, when a larger biopsy of the residual lesion was repeated, the pathologic evaluation established the diagnosis of an invasive squamous cell carcinoma adjacent to a verruca. Therefore, if the subsequent clinical features of a biopsy-proven wart do not correlate with the diagnosis of a benign verruca, additional evaluation should be considered to either confirm the existing diagnosis or establish a new diagnosis.

## Case presentation

A 63-year-old man presented for evaluation of a persistent lesion on his left hand of nine months duration. He recalls injuring the dorsum of his hand when he received a cut after a wrench fell on the injured site as he was repairing his motorcycle. He would repeatedly remove the scab; the lesion did not heal and progressively became larger. His past medical history was significant for chronic back pain (and one prior back surgery), cholecystectomy, hepatitis C (treated with eight weeks of ledipasvir/sofosbuvir), intravenous heroin use (previously and currently on daily methadone), and tobacco smoking.

**Figure 1:**
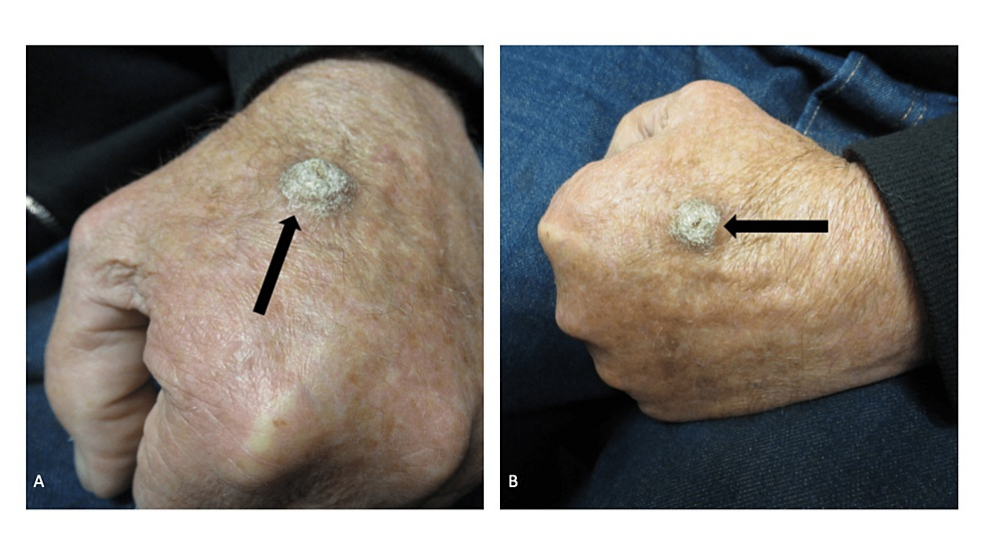
Verruca presenting as a nodule on the dorsal left hand A frontal view (A) and a superior view (B) of the dorsal left hand of a 63-year-old man shows a persistent, 18 by 18-millimeter verrucous hyperpigmented nodule (black arrow) of nine months duration. In addition to a wart, the clinical differential diagnosis included dermatofibroma, infection (such as bacterial, fungal, or mycobacterial), prurigo nodularis, and other possibilities such as a benign or malignant tumor. A biopsy, using the punch technique, was performed not only for microscopic examination but also for bacterial, fungal, and mycobacterial cultures.

Examination of his dorsal left hand showed an 18 by 18-millimeter verrucous hyperpigmented nodule (Figure 1); it was tender when pressure was applied to it. The clinical differential diagnosis, listed alphabetically, included dermatofibroma, infection (such as bacterial, fungal, or mycobacterial), ‘other’, prurigo nodularis, and verruca. A four-millimeter punch biopsy was performed for pathology evaluation; the biopsy tool was also used to remove a two-millimeter tissue specimen at the wound edge that was sent for bacterial, fungal, and mycobacterial cultures.

**Figure 2:**
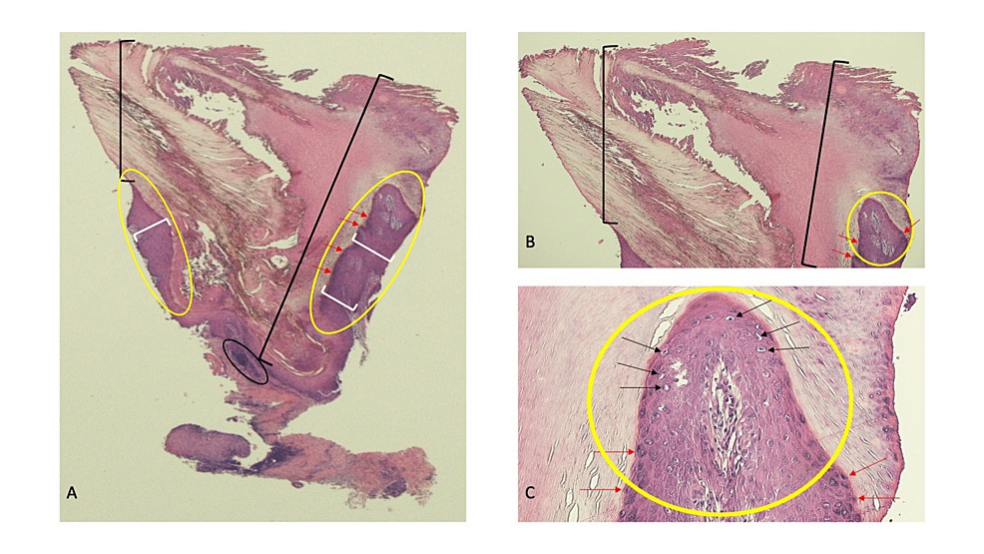
Pathologic presentation of a verruca on the dorsal hand of a 63-year-old man Distant (A) and closer (B and C) views of the microscopic features of the tissue specimen from the nodule on the patient’s dorsal hand demonstrated the pathologic changes of a wart. The epidermis showed massive thickening of the stratum corneum (hyperkeratosis, black brackets), thickening of the entire epidermis (acanthosis, white brackets), thickening of the granular layer (hypergranulosis, red arrows), an undulating configuration of the epidermis (papillomatosis, within the yellow ovals), and vacuolated keratinocytes (koilocytes, black arrows). In the upper dermis there is a band-like (lichenoid) inflammatory infiltrate (within the black oval) and in the dermal papillae there are numerous endothelial-lined vessels; some of the vessels contain red blood cells (hematoxylin and eosin stain: A, x2; B, x4; C, x 40).

Microscopic examination of the tissue specimen showed massive hyperkeratosis; in addition, the epidermis showed acanthosis, hypergranulosis, papillomatosis, and vacuolated keratinocytes (Figure 2). Vessels lined by endothelial cells, some of which contained erythrocytes, were in the dermal papillae. A lichenoid inflammatory infiltrate was also present in the upper dermis. These were the pathologic features of a wart; destructive (liquid nitrogen cryotherapy) or antiproliferative (topical 5-fluorouracil 5% cream) therapy was being considered for the treatment of the residual lesion.

The bacterial culture from the tissue specimen demonstrated moderate growth of *Staphylococcus intermedius*; the organism was susceptible to tetracycline. The possibility that the bacterial organism might be a non-pathogen was considered; however, the patient concurred with conservative management and was treated with doxycycline monohydrate 100 milligrams twice daily for 10 days. The tissue cultures for fungi and mycobacteria did not grow any organisms.

**Figure 3:**
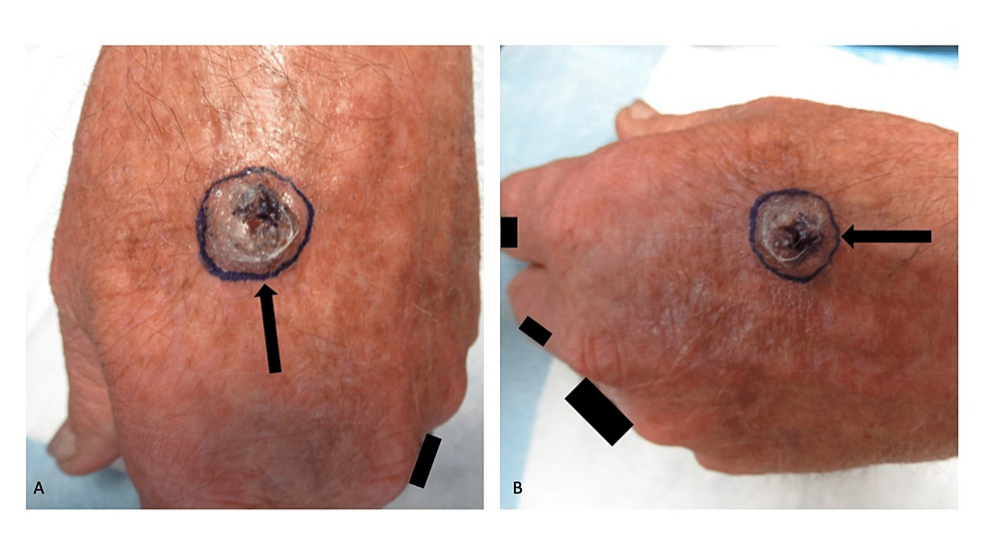
Squamous cell carcinoma with coexisting verruca, previously diagnosed by biopsy as a wart, presenting as a crusted nodule on the dorsal left hand A frontal view (A) and a superior view (B) of the dorsal left hand of a 63-year-old man, two weeks after being biopsied, showed a crusted nodule (black arrow) that had increased in both height and diameter; the solid black rectangles are present to obscure a ring on the fourth digit and small tattoos on the dorsal second and third digits. Only the tissue culture for bacteria grew an organism (*Staphylococcus intermedius*) which had been treated for 10 days with doxycycline monohydrate at a dose of 100 milligrams twice daily. Although the initial biopsy had established the diagnosis of a verruca, the current lesion had grown and did not have the typical morphology of a wart; indeed, the clinical differential diagnosis now included squamous cell carcinoma. Therefore, using the shave technique, an excisional biopsy (whose peripheral borders are demarcated by the purple-inked oval) was performed.

The patient returned for a follow-up after two weeks; he had completed his oral antibiotic treatment. The nodule on his left dorsal hand had increased in both height and diameter (Figure 3). Also, the central portion of the lesion did not have clinical features typically observed in a wart. The clinical differential diagnosis of the residual lesion included squamous cell carcinoma and verruca; there were no palpable axillary lymph nodes. An excisional biopsy using a shave technique was performed; hemostasis was achieved with electrodesiccation.

**Figure 4:**
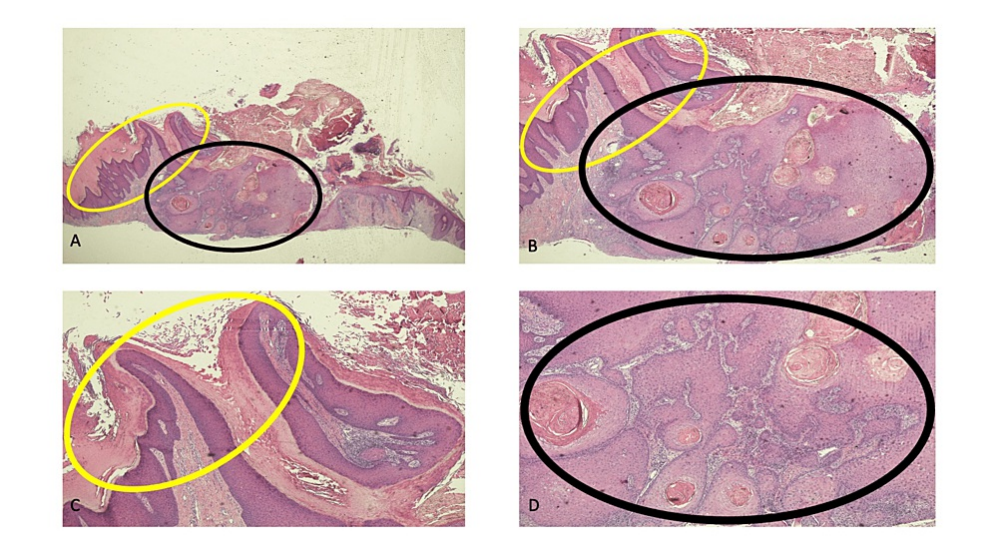
Pathologic presentation of a coexisting cutaneous squamous cell carcinoma and adjacent verruca on the dorsal hand of a 63-year-old man Distant (A) and closer (B, C, and D) views of the central portion of the specimen show the microscopic features of an invasive squamous cell carcinoma in the lesion (within the black oval). There is peripheral fibrosis surrounding the endophytic malignant tumor that consists not only of a proliferation of keratinocytes with hyperchromatic and pleomorphic nuclei but also small abscesses; the neoplasm that extends into the papillary and reaches the upper reticular dermis at the base of the biopsy specimen. In addition, in the lateral portion of the lesion (within the yellow oval), the microscopic features of a verruca are present; the exophytic benign wart has the following features: hyperkeratosis, mild acanthosis, papillomatosis, and endothelial-lined vessels in the dermal papillae (hematoxylin and eosin stain: A, x2; B, x4; C, x10, and D, x10).

Microscopic examination of the tissue specimen showed an epithelial neoplasm (Figure 4). The lateral portion of the specimen had exophytic warty features consisting of hyperkeratosis, papillomatosis, mild acanthosis, and endothelial-lined vessels in the dermal papillae. The central portion of the specimen consisted of an endophytic tumor characterized by proliferation of keratinocytes with hyperchromatic and pleomorphic nuclei that extended into the papillary and upper reticular dermis; small abscesses and peripheral fibrosis were also present. These were the pathologic changes of a verruca (at the periphery of the lesion) and an invasive squamous cell carcinoma (in the center of the lesion). 

The residual cutaneous squamous cell carcinoma was excised using the Mohs surgical technique. After clear margins were obtained, the surgical wound was closed by suturing the wound edges together. Follow-up examination after two weeks showed complete healing; at the subsequent follow-up visit three months later, there had been no recurrence of the tumor.

## Discussion

There are over 200 subtypes of human papillomavirus. Based on their phylogenetic characteristics, they have been divided into five genera: alpha, beta, gamma, mu, and nu. Alpha human papillomavirus predominantly infects mucosal epithelia; however, it has been associated with benign skin lesions and cutaneous warts. In contrast, beta human papillomavirus preferentially infects cutaneous sites [1,7].

Immunosuppression, whether congenital or acquired, increases an individual’s susceptibility to human papillomavirus infection. There are several inherited immunodeficiencies in which patients affected by the conditions have demonstrated an increased development of warts: ataxia telangiectasia, DOCK8 (dedicator of cytokinesis 8) deficiency causing a hyperimmunoglobulin E syndrome, epidermodysplasia verruciformis, GATA2 (GATA binding factor 2) deficiency, IL2RG (interleukin 2 receptor subunit gamma) deficiency, JAK3 (Janus kinase 3) deficiency, LAD1 (leucocyte adhesion deficiency 1), Netherton syndrome, severe combined immunodeficiency, NEMO (nuclear factor kappa-B essential modulator) deficiency syndrome, T-cell CD28 (cluster of differentiation 28) deficiency, WHIM (warts, hypogammaglobulinemia, infections, and myelokathexis) syndrome, and Whiskott-Aldrich syndrome. Similarly, increased human papillomavirus susceptibility has been associated with conditions and medications associated with acquired immunodeficiency including biologic therapy (such as tumor necrosis factor-alpha inhibitors), chemotherapy, connective tissue diseases (such as rheumatoid arthritis and systemic lupus erythematosus), human immunodeficiency virus infection, immunosuppressive agents (in solid organ transplant recipients), malignancy, and voriconazole [1,7,8].

There are several potential treatment options for verrucae. Although spontaneous resolution of warts (ranging as high as 60 percent to 70 percent within two years) has been observed, most patients prefer an active intervention. Common management approaches for treating a verruca can either be destructive (cantharidin, citric acid, cryotherapy, electrosurgery, formic acid, glycolic acid, laser surgery, monochloroacetic acid, phenol, pyruvic acid, salicylic acid, silver nitrate, surgical excision, trichloroacetic acid, and zinc oxide), immune modulating (interferon, intralesional candida antigen, Th-1 (T helper type 1) stimulating vaccination, and topical imiquimod), antiproliferative (bleomycin, 5-fluorouracil, podophyllin, podophyllotoxin, and vitamin D analogues), or antiviral (cidofovir and retinoids). Researchers have also suggested to consider evaluating a patient for immunodeficiency if they have recalcitrant warts, despite treatment with two or more modalities, that persist for more than 18 months [1,2]. In addition, it might be reasonable to consider a biopsy of the clinically suspected verruca in a patient whose lesion is chronic and/or fails to respond to therapy.

In addition to warts, human papillomavirus has been detected in benign neoplasms, premalignant lesions (such as actinic keratoses), and malignant tumors [7,9-17]. A study of 51 non-genital seborrheic keratoses observed that two acanthotic seborrheic keratoses were positive for alpha human papillomavirus deoxyribonucleic acid (DNA): high-risk human papillomavirus 31 and low-risk human papillomavirus 42 [16]. A 76-year-old woman developed a new exophytic flesh-colored verrucous nodule on her left plantar hallux that had been enlarging for several months; a biopsy revealed a combined lesion consisting of a verruca plantaris (which demonstrated strongly positive staining with p16 in the epidermis correlating with human papillomavirus infection) and a poroma (which showed positive staining with both carcinoembryonic antigen and epithelial membrane antigen) [17].

Human papillomavirus has been shown in basal cell carcinomas [7,9,10]. Beta human papillomavirus was detected in 17 percent (16 of 96) of basal cell carcinomas studied from non-epidermodysplasia verruciformis patients [7]. Other investigators, evaluating skin swabbings, also demonstrated beta human papillomavirus on the skin of patients who developed basal cell carcinoma [9,10]. In addition, one of the studies also showed that the risk of developing basal cell carcinoma may increase with increased numbers of beta human papillomavirus subtypes on the skin [10].

Cutaneous squamous cell carcinoma in situ may present as a superficial, scaly red plaque that clinically mimics cutaneous basal cell carcinoma in situ or dermatitis. Cutaneous squamous cell carcinoma can present as a plaque or nodule with either an intact or ulcerated surface; it is usually located on a sun-exposed site. Several clinical and pathologic parameters have been assessed to stratify the tumor as either being high-risk or low-risk for local recurrence, nodal and distant metastases, and disease-specific death [3,4,18].

The association of beta human papillomavirus, ultraviolet radiation exposure, and the development of cutaneous squamous cell carcinoma was initially observed in patients with the autosomal recessive genodermatosis epidermodysplasia verruciformis. Early in life, patients with epidermodysplasia verruciformis present with flat warts that are disseminated, persistent, and associated with beta human papillomavirus five and beta human papillomavirus eight. Subsequently, they are prone to develop ultraviolet radiation-induced cutaneous squamous cell carcinomas [5,6]. 

Solid organ transplant patients also have an increased susceptibility to beta human papillomavirus infection. The observation of cutaneous squamous cell carcinoma in non-epidermodysplasia verruciformis solid organ transplant patients supported the hypothesis that beta human papillomavirus has a carcinogenic role in cutaneous squamous cell carcinoma in patients with other etiologies for their immunosuppression. Based on these observations, investigators consider human papillomavirus and ultraviolet radiation to be cocarcinogens in the development of cutaneous squamous cell carcinoma [7,9,10].

Subsequently, documentation of human papillomavirus infection has been observed not only in immunosuppressed patients but also in immunocompetent individuals who developed either cutaneous squamous cell carcinoma in situ or squamous cell carcinoma [7,9,10,19,20]. Beta human papillomavirus was detected in 100 percent (four of four) of cutaneous squamous cell carcinomas from three epidermodysplasia verruciformis patients. Beta human papillomavirus was also detected in 31 percent (23 of 74) of cutaneous squamous cell carcinomas from non-epidermodysplasia verruciformis patients [7].

The risk of developing cutaneous squamous cell carcinoma is increased by the presence of beta human papillomavirus. However, when baseline cutaneous human papillomavirus was evaluated, less than five percent of beta human papillomavirus types originally detected in the skin swabs were subsequently found to be present in the cutaneous squamous cell carcinoma. The investigators concluded that although the detection of beta human papillomavirus in skin swabs was a significant predictor of cutaneous squamous cell carcinoma, only a small number of the tumors were etiologically linked to the viral infection [9,10].

There are also several individual reports or case series of patients with cutaneous squamous cell carcinoma in which human papillomavirus has been documented. These have occurred at locations on the head including the scalp and ear [11,12]. They have also been shown to develop on the distal upper extremity, such as the hands, fingers, and nail unit [13-15].

A case series of seven men (ranging in age from 71 years to 83 years; median, 78 years) and a woman (78 years old) described nine cutaneous squamous cell carcinomas of the scalp. The carcinomas demonstrated either one (three tumors), two (two tumors), or three (four tumors) subtypes of beta human papillomavirus. The investigators postulated that the development of the scalp cutaneous squamous cell carcinomas occurred secondary to a synergistic effect of ultraviolet radiation and the human papillomavirus gene expression [11]. 

A 73-year-old man presented with a crater-like lesion of his left conchal bowl of two months duration; although a squamous cell carcinoma was clinically suspected, the initial biopsy only showed an epidermoid cyst with human papillomavirus-induced (p16 positive staining) changes. The lesion persisted and a second biopsy of the lesion was performed; this showed an invasive cutaneous squamous cell carcinoma and additional evaluation demonstrated that the tumor not only involved the external auditory canal, but also the temporomandibular joint and parotid gland. Hence, similar to the dorsal hand lesion of the 63-year-old patient described in this report, the man’s ear lesion concurrently contained both a cutaneous squamous cell carcinoma and a human papillomavirus-associated benign lesion [12].

Also similar to the coexisting squamous cell carcinoma and adjacent verruca on the hand of the man in this report, a 59-year-old woman (previously treated with azathioprine for more than 20 years and currently receiving glatiramer acetate for multiple sclerosis) presented with a lesion on her dorsal hand: a scaly erythematous patch. Microscopic examination showed a cutaneous squamous cell carcinoma in situ. Additional studies demonstrated that the lesion also contained alpha human papillomavirus 67 [13].

Human papillomavirus-associated digital cutaneous squamous cell carcinoma in situ or squamous cell carcinoma has been described on the fifth digit in two immunocompetent patients: a 64-year-old woman and a 68-year-old man [13,14]. The woman’s painful and aesthetically disturbing tumor had been present for five years as a slightly erythematous, keratotic papule which had clinically been interpreted as a wart and twice unsuccessfully treated with cryotherapy using liquid nitrogen prior to a biopsy that established the correct diagnosis; alpha human papillomavirus 73 was discovered in the in situ carcinoma and the cancer site was completely excised without recurrence of the tumor during six years of follow-up [14]. The man’s invasive carcinoma presented as a verrucous hyperkeratotic plaque; alpha human papillomavirus 67 was detected in the tumor [13].

Human papillomavirus-associated squamous cell carcinoma in situ and squamous cell carcinoma have been demonstrated in ungual and periungual tumors. Only seven percent of the patients were immunosuppressed. The patients ranged in age from 22 to 89 years; the tumors affected men twice as often as women, and the neoplasms presented as persistent verrucae that had been present for an average of 5.3 years. Alpha human papillomavirus 16 was the most common subtype identified in nearly 75 percent of the cases; however, several other alpha human papillomavirus subtypes were also found: 2, 11, 18, 26, 31, 35, 56, 58, and 73. In comparison to cutaneous squamous cell carcinoma in other sites, these tumors had a higher post-excision recurrence rate; also, digital amputation was required for six percent of the cases [15].

The initial clinical features of the reported patient’s hand nodule were compatible with a verruca. Indeed, the microscopic evaluation of the punch biopsy specimen had pathologic features of a verruca and thereby confirmed the suspected diagnosis. However, the lesion not only persisted but increased in size after the biopsy. Therefore, additional evaluation (including a larger specimen providing more tissue for examination) was performed. The second biopsy not only showed features of a benign verruca at the periphery of the lesion, but also an invasive squamous cell carcinoma in the center of the nodule. Correlating the sequence of clinical presentations and pathologic evaluations, we consider the patient’s dorsal hand nodule to be a coexistent verruca and squamous cell carcinoma and speculate that the verruca may have been etiologically related to the pathogenesis of the cutaneous squamous cell carcinoma.

## Conclusions

Human papillomavirus infection has not only been demonstrated in verruca, but also in other benign skin tumors (including epithelial lesions such as seborrheic keratoses and adnexal tumors such as poromas), precancerous lesions such as actinic keratoses, and cutaneous malignancies such as nonmelanoma skin cancers. Risk factors for the pathogenesis of cutaneous squamous cell carcinoma include chronic exposure to ultraviolet radiation and immunosuppression; in addition, human papillomavirus infection has been shown to be a cocarcinogen with ultraviolet radiation in the carcinogenesis of this neoplasm. A 63-year-old man developed a verrucous nodule on his dorsal hand. A small punch biopsy confirmed the clinically suspected diagnosis of a verruca. However, the possibility of a squamous cell carcinoma was considered when the lesion continued to grow. The microscopic examination of a larger shave biopsy of the residual lesion showed both a benign verruca at the periphery of the lesion and an invasive squamous cell carcinoma in the central portion of the nodule. It is reasonable to postulate, based on the observation of the coexisting benign wart adjacent to the malignant skin cancer, that the patient’s human papillomavirus-associated verruca may have contributed to the development of his invasive cutaneous squamous cell carcinoma. In conclusion, the possibility of an alternative or concurrent tumor, such as a cutaneous squamous cell carcinoma, should be entertained in a patient in whom a verruca, suspected clinically or confirmed pathologically, does not resolve following wart-directed therapy.
